# Bioinspired Precision Peeling of Ultrathin Bamboo Green Cellulose Frameworks for Light Management in Optoelectronics

**DOI:** 10.1007/s40820-025-01867-1

**Published:** 2025-08-05

**Authors:** Yan Wang, Yuan Zhang, Yingfeng Zuo, Dawei Zhao, Yiqiang Wu

**Affiliations:** 1 College of Materials and Energy, Central South University of Forestry and Technology, Changsha, 410004 Hunan People’s Republic of China; 2 Key Laboratory On Resources Chemicals and Materials of Ministry of Education, Shenyang University of Chemical Technology, Shenyang, 110142 Liaoning People’s Republic of China

**Keywords:** Bamboo green, Cellulose framework, Chemical peeling, Optical properties, Light management

## Abstract

Cellulose frameworks have emerged as promising materials for light management due to their exceptional light-scattering capabilities and sustainable nature. Conventional biomass-derived cellulose frameworks face a fundamental trade-off between haze and transparency, coupled with impractical thicknesses (≥ 1 mm). Inspired by squid’s skin-peeling mechanism, this work develops a peroxyformic acid (HCOOOH)-enabled precision peeling strategy to isolate intact 10-µm-thick bamboo green (BG) frameworks—100 × thinner than wood-based counterparts while achieving an unprecedented optical performance (88% haze with 80% transparency). This performance surpasses delignified biomass (transparency < 40% at 1 mm) and matches engineered cellulose composites, yet requires no energy-intensive nanofibrillation. The preserved native cellulose I crystalline structure (64.76% crystallinity) and wax-coated uniaxial fibril alignment (Hermans factor: 0.23) contribute to high mechanical strength (903 MPa modulus) and broadband light scattering. As a light-management layer in polycrystalline silicon solar cells, the BG framework boosts photoelectric conversion efficiency by 0.41% absolute (18.74% → 19.15%), outperforming synthetic anti-reflective coatings. The work establishes a scalable, waste-to-wealth route for optical-grade cellulose materials in next-generation optoelectronics.

**Supplementary Information:**

The online version contains supplementary material available at 10.1007/s40820-025-01867-1.

## Highlights

- First successful peeling of bamboo green into micrometer-scale optical films (10 μm) via a bioinspired peroxyformic acid strategy, achieving intact preservation of monolayer cellular structure.
- Scalable and stable peeling process enables high-yield production of bamboo green frameworks, demonstrating significant potential for sustainable optical material applications.
- Experimental validation in light management shows 0.41% absolute photoelectric conversion efficiency enhancement in solar cells, proving practical value as high-performance optical films.

**Supplementary Information:**

The online version contains supplementary material available at 10.1007/s40820-025-01867-1.

## Graphical abstract

**Figure:**
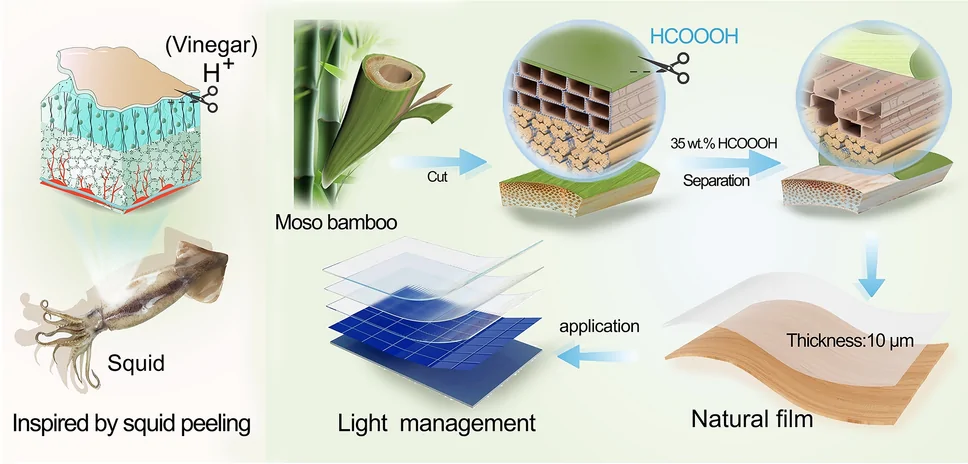

## Introduction

While Moso bamboo (*Phyllostachys edulis*) has been extensively utilized as a structural material, its outermost layer—the bamboo green (BG)—remains largely unexplored despite possessing unique structural merits. Distinct from the well-characterized fibrous structure of inner bamboo timber, BG exhibits a hierarchically layered cellular organization with 6–7 layers (total thickness: 110–116 μm) [1], combining mechanical flexibility unmatched by bulk wood-derived cellulose frameworks. Although current industrial processing typically discards BG as a by-product due to its waxy surface chemistry (which impedes adhesive bonding in composites) [2], this biomass possesses underutilized photonic potential from its air-cellulose interfaces. For reference, delignified balsa wood exhibits a characteristic refractive index of 1.5 [3]. Critically, the absence of reported research for isolating intact BG frameworks reflects a fundamental challenge: Existing methods invariably degrade either the cellular integrity or native cellulose alignment essential for functionality.

High-haze transparent materials are transforming optoelectronic and energy technologies by uniquely reconciling two traditionally antagonistic properties: exceptional transparency (transparency > 80%) and precisely tunable light scattering (haze > 70%). These advanced optical materials address critical challenges in next-generation devices, including photovoltaics [4], flexible displays [5, 6], and architectural smart windows [7]. However, current industry solutions—including laser-etched silica glass and nanoparticle-embedded polymers—fundamentally suffer from inherent trade-offs between optical clarity and haze control. Meanwhile, emerging biobased alternatives face scalability challenges due to their insufficient optical transparency and energy-intensive processing [8–10]. Bulk biomass-derived frameworks (e.g., wood, bamboo timber) achieve > 80% haze but suffer from opacity (transparency < 40%) at practical thicknesses (≥ 1 mm), while bottom-up nanocellulose films—though optically tunable—require energy-intensive disintegration and multistep processing (e.g., TEMPO oxidation [11], vacuum filtration [12]) that disrupt native fibril frameworks. Macroscale biomass matrices rely on original fibril entanglement for haze [13], but this introduces Mie scattering losses that limit transparency. Nanocellulose assemblies (< 100 nm fibrils) reduce scattering via Rayleigh regimes, yet their restacked architectures sacrifice the natural hierarchical porosity essential for broadband light management [14]. Recent work has attempted to bridge this gap through hybrid composites (e.g., CNC/PVA), but these demand synthetic polymer binders that undermine sustainability [15, 16].

**Fig. 1:**
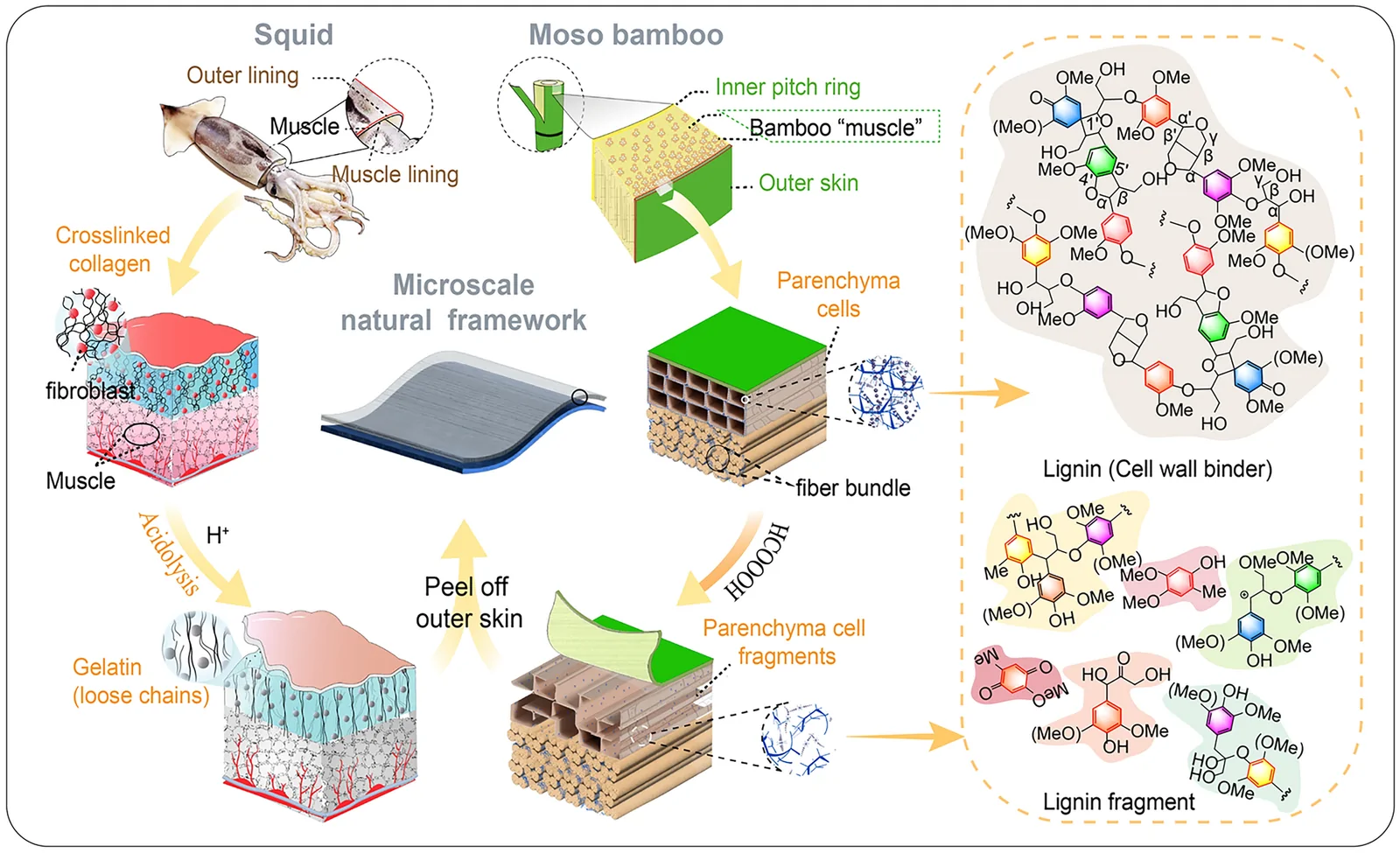
Intact bamboo green framework isolation achieved through squid skin-muscle inspired peeling

Inspired by the acetic acid-assisted peeling of squid mantle skin in food preparation, a similar “skin-muscle” structure in Moso bamboo was noticed (Fig. 1). In squid mantle, the skin is connected to underlying muscle through collagen-rich connective tissue, where acetic acid in food vinegar selectively disrupt the cross-links of collagen chain while preserving the skin’s structural integrity [17]. This biological paradigm inspired our development of a chemical approach for isolating intact BG cellular frameworks (Fig. 1). As a comparison, the bamboo “muscle” consists mainly of fiber bundles connected to the BG through parenchyma cells [18]. These parenchyma cells are primarily composed of cellulose, hemicelluloses, and lignin, with lignin playing an adhesive role. Therefore, the destruction to parenchyma cell layers and three cross-linking components in its cell wall facilitates the peeling of BG. Existing research primarily focuses on delignification as a destructive approach to plant cell structures, which involves significant depolymerization of lignin itself [19–21]. Developing nondestructive methods for precise cell layer separation—a longstanding obstacle in biomass utilization—forms the central innovation of this work.

Herein, this work pioneers a waste-to-optical-material approach by repurposing bamboo green—an industrial by-product—into ultrathin high-performance frameworks via a mild HCOOOH peeling process. Unlike conventional delignification, this peeling strategy selectively targets parenchyma cell junctions, preserving the native architecture (cellulose I crystallinity: 64.76%) and enabling 88% haze with 80% transparency at merely 10 μm thickness. The frameworks’ bidirectional wettability (wax-coated outer surface vs. hydrophilic inner layer) and mechanical robustness (903 MPa modulus) facilitate its integration as light-management layer, achieving a 0.41% absolute photoelectric conversion efficiency (PCE) boost in silicon photovoltaics—a milestone for sustainable optical materials.

## Experimental Section

### Materials and Chemicals

2–3-year-old Moso bamboo (*Phyllostachys edulis*) culms (8–12 cm diameter) were obtained from Hunan Taohuajiang Bamboo, Co., Ltd. (China). Poplar (*Populus tomentosa*) sheets (3–4 mm thickness) and bamboo timber slices (1–2 mm thickness) were sourced from Yiyang City, Hunan Province. For the bamboo green peeling process, this work used: hydrogen peroxide (> 30 wt%, H_2_O_2_), formic acid (> 88 wt%, HCOOH), and sulfuric acid (> 98 wt%, H_2_SO_4_). All chemicals were purchased from Sinopharm Group Chemical Reagent Co., Ltd. Commercial polycrystalline silicon solar panels (3 cm × 4 cm) were procured from Shenzhen Kobe Electronic Technology Co., Ltd.

### Preparation of Bamboo Green Framework

Raw bamboo culms were sawn into individual stems (without nodes) and cut longitudinally into bamboo strips, which were then soaked in water to remove water-soluble sugars and extracts. Bamboo strips (8 cm × 3 cm × 0.8 cm wall thickness) were air-dried at 25–30 °C until the moisture content reached approximately 50%, followed by drying at 50 °C until the moisture content was approximately 20%. The dried bamboo strips were treated using a peroxyformic acid solution. (H_2_O_2_ and HCOOH were mixed at molar ratios of n(H_2_O_2_):n(HCOOH) = 2:1, 1.5:1, 1:1, and 1:2 for reaction.) H_2_SO_4_ was used as the catalyst, added at 0.5 wt% of the total solution mass. The peroxyformic acid solution was added at different dosages (with 10 g of solution per 1 g of bamboo sample defined as 1 portion). A constant-temperature water bath was preheated to the set reaction temperatures (60, 65, 70, and 75 °C). After mixing the bamboo strips with the peroxyformic acid solution, the mixture was placed in the water bath for reaction. Once the bamboo green peeled to form a framework, heating was stopped, and the peeling time was recorded. After being washed multiple times with deionized water, the framework was preserved in anhydrous ethanol. Control samples (poplar sheets and bamboo timber slices) were processed identically until complete whitening occurred.

### Characterization Methods

Frameworks thickness was measured using an AO-HK830-5870 stereomicroscope. Morphology was examined by scanning electron microscopy (SEM, MIRA3 TESCAN) with energy-dispersive X-ray spectroscopy (EDS) for elemental analysis. Crystalline structure was determined by X-ray diffraction (XRD, D8 ADVANCE, Bruker; Cu Kα radiation, *λ* = 1.54 Å, 40 kV). Molecular changes were analyzed by Fourier transform infrared spectroscopy (FTIR, Spotlight 400; 4000 − 600 cm^−1^ range) and X-ray photoelectron spectroscopy (XPS, Kratos AXIS SUPRA + ; monochromatic Al K*α* source). Transmittance and haze were measured using the UV-integrating sphere (UV-3150 Shimadzu, Japan, LISR-3100, 5 − 95% haze range) and the LAMBDA 950 UV/Vis/NIR spectrophotometer (PerkinElmer, USA). The wide-angle X-ray scattering (WAXS) measurements were conducted on a Xeuss 2.0 WAXS system with a Cu Kα (*λ* = 1.54189 Å) microfocus source and Dectris Pilatus 3R 300 K detector in transmission mode in vacuum. Tensile tests were evaluated per GB 13022-1991 standard (MTS system, 0.5 mm min^–1^ crosshead speed). The *J–V* characteristics and PCE of the solar cells were measured under AM 1.5G illumination (CEL-QPCE1000 system, CEAuLight). For statistical validation, three independent solar cells were fabricated and tested 5 times for each group (original cell, EVA-only encapsulated cell, and EVA-BG encapsulated cell). All reported photovoltaic parameters represent mean values ± standard deviation derived from these replicates. Significant differences were detected by one-way ANOVA. Additionally, the biomass composition (cellulose, hemicellulose, and lignin) was quantified using the NREL Laboratory Analytical Procedure, while the cellulose crystallinity index (*CrI*) was calculated via the Turley–Segal method based on XRD data: *CrI* (%) = (*I*_002_ − *I*_amorph_)/*I*_002_ × 100, where *I*_002_ is the peak intensity of the (002) lattice plane (2*θ* ≈ 22.5°), and *I*_amorph_ represents the minimum intensity between the (101) and (002) peaks (2*θ* ≈ 18°), corresponding to the amorphous region.

## Results and Discussion

### Precision Peeling of Bamboo Green: Mechanism and Framework Preservation

#### Mechanistic Foundations of Cellular Disconnection

**Fig. 2:**
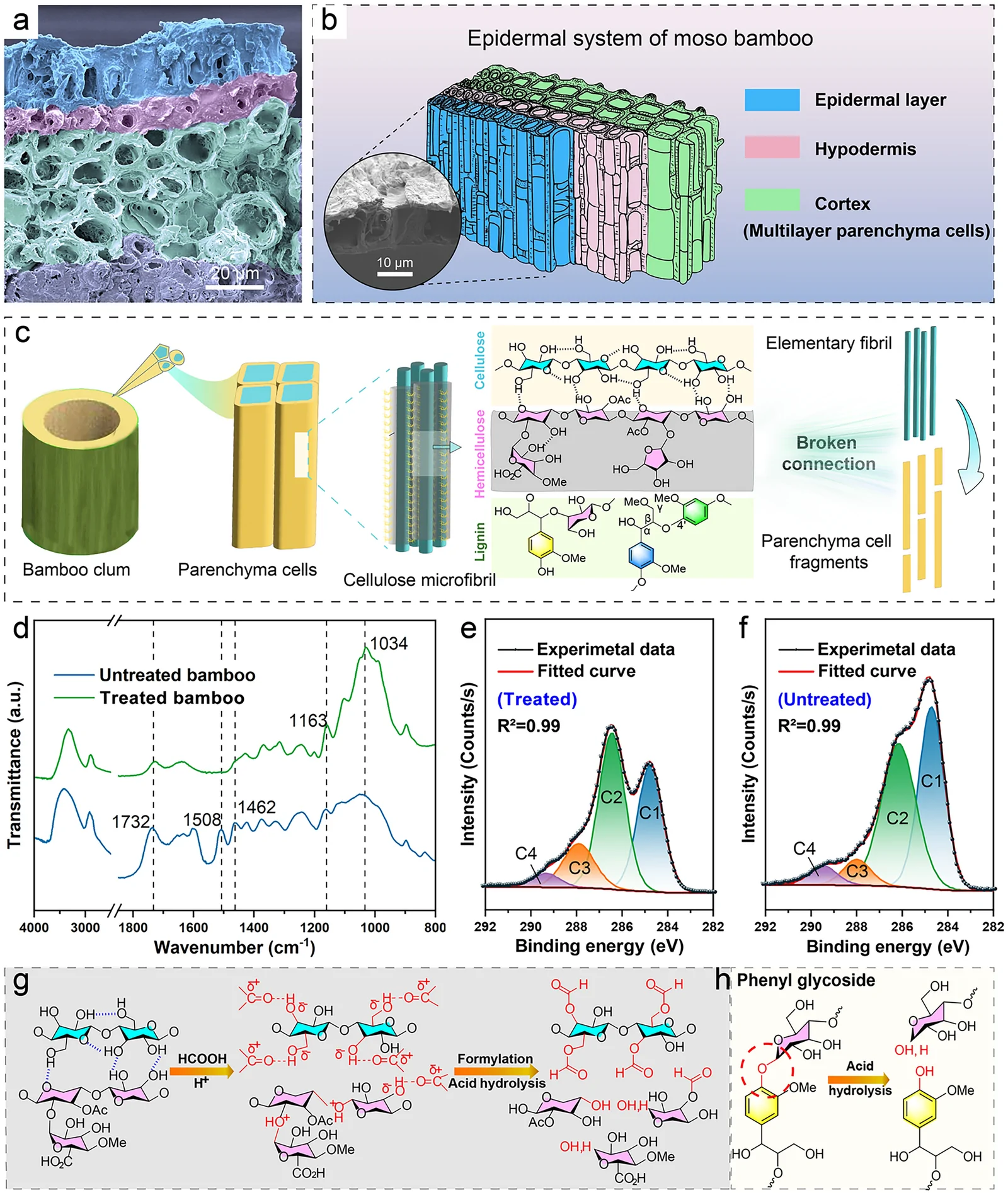
**a** SEM image and **b** three-dimensional schematic diagram of Moso bamboo epidermis system. **c** Illustration of parenchyma cells disruption from the cellular to molecular level. **d** Comparative FTIR analysis of untreated versus treated bamboo samples. High-resolution C 1*s* XPS spectra of **e** treated and **f** untreated bamboo samples. **g** Hydrogen-bond breaking reaction resulting from the acetylation of cellulose and hemicellulose. **h** Hydrolysis of the phenyl glycoside bond between hemicellulose and lignin

High-resolution scanning electron microscopy (SEM) imaging reveals the distinct trilaminar organization of bamboo green (BG), comprising (Fig. 2a, b): An epidermal layer with thick cuticular deposits, a hypodermis characterized by reinforced cell walls, and a cortex primarily composed of parenchyma cells. These parenchyma cells within the cortex, which directly interconnect with the fiber bundles in the bamboo “muscle,” are characterized by abundant longitudinal pits (Fig. S1), relatively thinner cell walls, and significantly larger volumes compared to cells in the epidermal and hypodermal layers (Fig. 2a). This unique structural configuration creates preferential reagent penetration pathways, with ~ 71% surface porosity (vs. 50.5%–20.3% in vascular bundles, Fig. S1) [22], rendering the parenchyma cells substantially more susceptible to chemical treatments than the adjacent robust tissues. Such pronounced structural disparity provides an exceptional opportunity for selective removal of parenchyma cells while preserving the structural integrity of the surrounding tissues.

The destruction of parenchyma cells within the cortex primarily results from the breakdown and dissolution of cellulose, hemicellulose, and lignin in their cell walls. As illustrated in Fig. 2c, hemicelluloses coat on the surface of cellulose microfibrils and are connected to cellulose through hydrogen bonding between hydroxyl groups [23]. Meanwhile, lignin forms lignin–carbohydrate complexes (LCCs) with hemicelluloses through covalent bonds [24]. From a “disconnection” perspective, the disintegration of the cortex is driven by two key mechanisms: impairment of cellular junctions and disruption of intercomponent bonds. Among delignification reagents, peroxyformic acid (HCOOOH) is considered an optimal chemical reagent for the BG peeling process, owing to its high reaction rate and abundance of active species. Highly selective hydroxyl cations (HO^+^), peracetate ions (HCOOO^–^), and sulfuric acid (H^+^) present in HCOOOH can collectively degrade lignin, hemicelluloses, and cellulose in both cell walls and middle lamella [25].

FTIR and XPS were employed to analyze bamboo samples before and after HCOOOH treatment (Fig. 2d–f). FTIR analysis revealed an obvious increase in peak intensity at 1034 cm^−1^ (C–O stretching vibration) and at 1163 cm^−1^ (C–O–C antisymmetric stretching vibration), indicating enhanced C–O–C bond formation (Fig. 2d). This phenomenon is attributed to the strong acylation capability of formate ions (HCOO^–^) in the HCOOOH solution under acidic catalysis [26]. During oxyacylation, hydroxyl groups (–OH) on cellulose and hemicellulose chains were selectively substituted by formyl groups (–CHO), thereby promoting C–O–C bond formation (Fig. 2g). XPS analysis demonstrated a marked increase in the O/C atomic ratio (Table S1) and enhanced peak intensities for C2 (C–O) and C3 (C = O) in the high-resolution C 1* s* spectrum (Fig. 2e, f). These findings confirm the oxyacylation reaction and elucidate HCOOOH’s dual role in disrupting interchain hydrogen bonds via hydroxyl group substitution.

The content of cellulose, hemicellulose, and lignin in bamboo samples was quantified using the National Renewable Energy Laboratory (NREL) standard method. Following HCOOOH treatment, the hemicellulose content decreased dramatically by 55% compared to untreated samples, while cellulose and lignin contents showed minimal changes (< 2 wt% reduction, Table S2). This selective removal indicates extensive solubilization of hemicellulose-derived monosaccharides into the solution. The degradation mechanism of hemicellulose involves: (i) Acid-catalyzed hydrolysis: Under acidic conditions, protonation of glycosidic atoms in hemicellulose polysaccharides induces charge separation, enabling nucleophilic attack by H^+^ ions that cleave glycosidic bonds (Fig. 2g) [27]. (ii) Xylan-specific reactions: As xylan comprises > 90% of bamboo hemicellulose, its decomposition dominates the process [28]. FTIR analysis confirmed this through diminished intensity at 1732 cm^−1^ (C = O stretching of xylan). (iii) Lignin–carbohydrate complex (LCC) cleavage: Among the acid-labile LCC linkages (*β*-aryl ether, acyl, and phenyl glycoside), the phenyl glycoside bonds between xylan and lignin are particularly susceptible to hydrolysis (Fig. 2h), as demonstrated in prior studies [28–30]. These concerted reactions disrupt cellulose-hemicellulose-lignin networks, inducing cell wall delamination and ultimately, structural collapse.

#### Multiscale Validation and Process Optimization

**Fig. 3:**
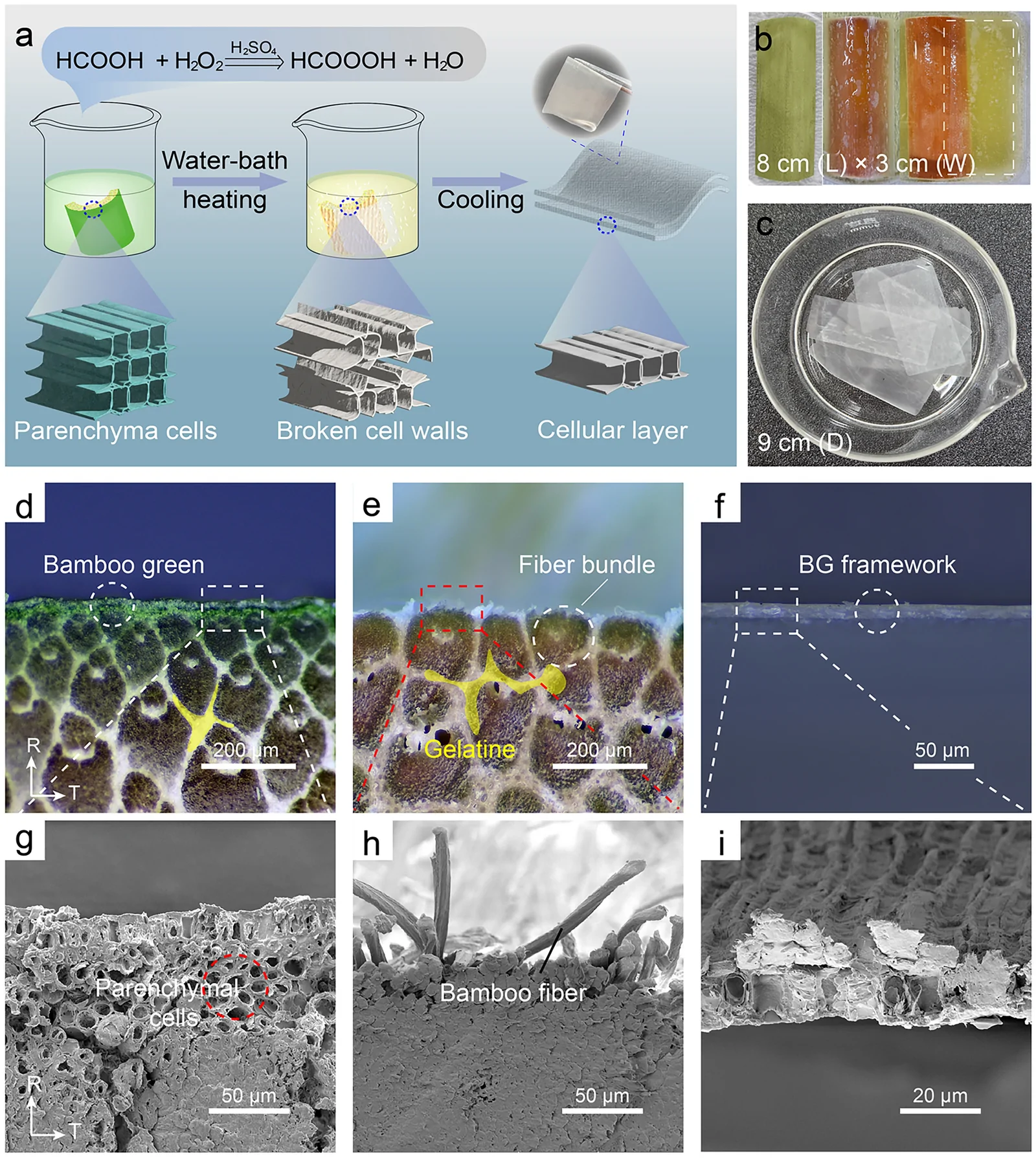
**a** Illustration of the preparation of BG framework. **b** Peeling of BG occurs in three states: initial morphology, partial peeling, and complete peeling. **c** BG frameworks in a 9 cm culture dish. **d-f** Optical and **g-i** SEM cross sections: untreated bamboo, peeled bamboo, and BG framework

In a standard experiment, bamboo strips (8 cm × 3 cm × 0.8 cm wall thickness) were immersed in HCOOOH reagent and heated in a water bath at mild temperature until complete BG peeling was achieved (Fig. 3a–c). This process results in the production of natural cellulose framework (Fig. 3b, c), demonstrating targeted destructive effects of HCOOOH on the connection layer—the parenchyma cells. Cross-sectional analysis of bamboo strips before and after HCOOOH treatment was conducted using stereomicroscope (XTX) and SEM (Fig. 3d–i). Prior to treatment, microscopic examination revealed a well-defined boundary between the BG and bamboo “muscle” (Fig. 3d), connected through intact parenchyma cell layers, with the bamboo “muscle” itself consisting of densely packed, well-organized fiber bundles (Fig. 3g). Following BG peeling, the fiber bundles maintained their original configuration and structural integrity (Fig. 3e), while the parenchyma cell layers were entirely removed, leaving behind the bare fiber bundles (Fig. 3h). The resulting peeled framework exhibited a distinct monolayer cellular structure (Fig. 3f, i), demonstrating the selective and precise action of the HCOOOH treatment on the parenchyma cell layer while preserving the surrounding tissue architecture.

Unlike conventional bottom-up pulping or top-down cellulose framework preparation, this peeling approach directly exfoliates the native microscale framework from BG. However, maintaining framework integrity and achieving complete peeling pose significant challenges, necessitating precise process control for reproducible results. Specifically, an excessively high peeling speed can induce surface tearing of the framework, whereas an insufficient peeling speed preserves surface integrity but prevents complete detachment from the bamboo strip. To optimize the process, the influence of four parameters—HCOOOH formulation ratio, HCOOOH concentration, reaction temperature, and solution dosage—on the peeling rate and vigor of reaction was investigated, utilizing peeling time (min, *t*) and mass loss rate (%, MLR) as the indicators for assessment. The synthesis of HCOOOH typically proceeds through a reversible reaction between hydrogen peroxide (H_2_O_2_) and formic acid (HCOOH), catalyzed by concentrated sulfuric acid (H_2_SO_4_) (Fig. 3a). While high temperatures (≥ 60 °C) accelerate HCOOOH production, they concurrently increase its thermal decomposition, rendering direct concentration measurements unreliable for kinetic studies. To overcome this limitation, HCOOOH generation was indirectly quantified through reaction exothermicity measurements. Temperature–time profiles (Fig. S2) were recorded for reaction solutions with varying H_2_O_2_:HCOOH molar ratios (2:1, 1.5:1, 1:1, and 1:2) in a water bath set at 60 °C. After initial equilibration to the bath temperature, the solutions exhibited further temperature increases due to reaction exothermicity. As shown in Fig. S2, the 1:1 molar ratio demonstrated optimal performance, with the solution temperature rapidly rising to 98 °C within 33 min, indicating efficient HCOOOH generation and ideal reagent dosage.

Single factor and response surface methodology (RSM) were utilized to thoroughly assess and optimize the other parameters: reaction temperature (45, 55, 65, and 75 °C; **B**), solution dosage (0.6, 0.8, 1.0, and 1.2 portions; **C**), and HCOOOH concentration (26%, 29%, 32%, and 35%; **D**) (Table S3, Fig. S3). At lower HCOOOH concentrations (26% and 29%), the bamboo samples showed an inadequate reaction (MLR < 7%), resulting in incomplete BG peeling. Moreover, effective peeling required temperatures higher than 65 °C (*t* < 30 min, 7% < MLR < 13.35%), and the solution dosage should be within the range of 0.8–1.2 portions. The validated response surface model (MLR = 3.8 + 1.89B + 0.2750C + 2.67D − 1.07BC − 0.5100BD − 0.0425CD + 1.24B^2^ + 0.5990C^2^ + 1.02D^2^) demonstrated excellent predictive accuracy (*p* < 0.0001), with parameter significance: D > B > C (*p* < 0.05 for B × C correlation) (Table 1). Validation at optimal conditions (35% HCOOOH, 68 °C, 1 portion) yielded 7.4% MLR (0.3% deviation from prediction), confirming model reliability for reproducible framework production (Table 2).

**Table 1:** Analysis of variance for the quadratic response surface model of bamboo mass loss rate

Source	Sum of squares	df	Mean square	*F*-value	*p*-value
Model	105.47	9	11.72	30.50	< 0.0001
B	28.54	1	28.54	74.28	< 0.0001
C	0.6050	1	0.6050	1.57	0.2498
D	56.98	1	56.98	148.30	< 0.0001
BC	4.60	1	4.60	11.98	0.0105 (< 0.05)
BD	1.04	1	1.04	2.71	0.1438
CD	0.0072	1	0.0072	0.0188	0.8948
B^2^	6.49	1	6.49	16.89	0.0045 (< 0.05)
C^2^	1.51	1	1.51	3.93	0.0878
D^2^	4.39	1	4.39	11.44	0.0117
Residual	2.69	7	0.3842		
Lack of fit	2.13	3	0.7093	5.05	0.0758
Pure error	0.5617	4	0.1404		
Cor total	108.16	16			

*p*-value < 0.05 (significant effect); *p*-value < 0.01 (highly significant effect)

**Table 2:** Verification of model predictions with experimental results

Factor value	B	C	D	Response variable
	Temperature (°C)	Dosage (P, Portion)	Concentration (%)	Mass loss rate (%)
Predicted	68.39	1.02	34.90	7.10
Actual validation	68	1.00	35	7.40

### Structure and Composition of Bamboo Green Framework

**Fig. 4:**
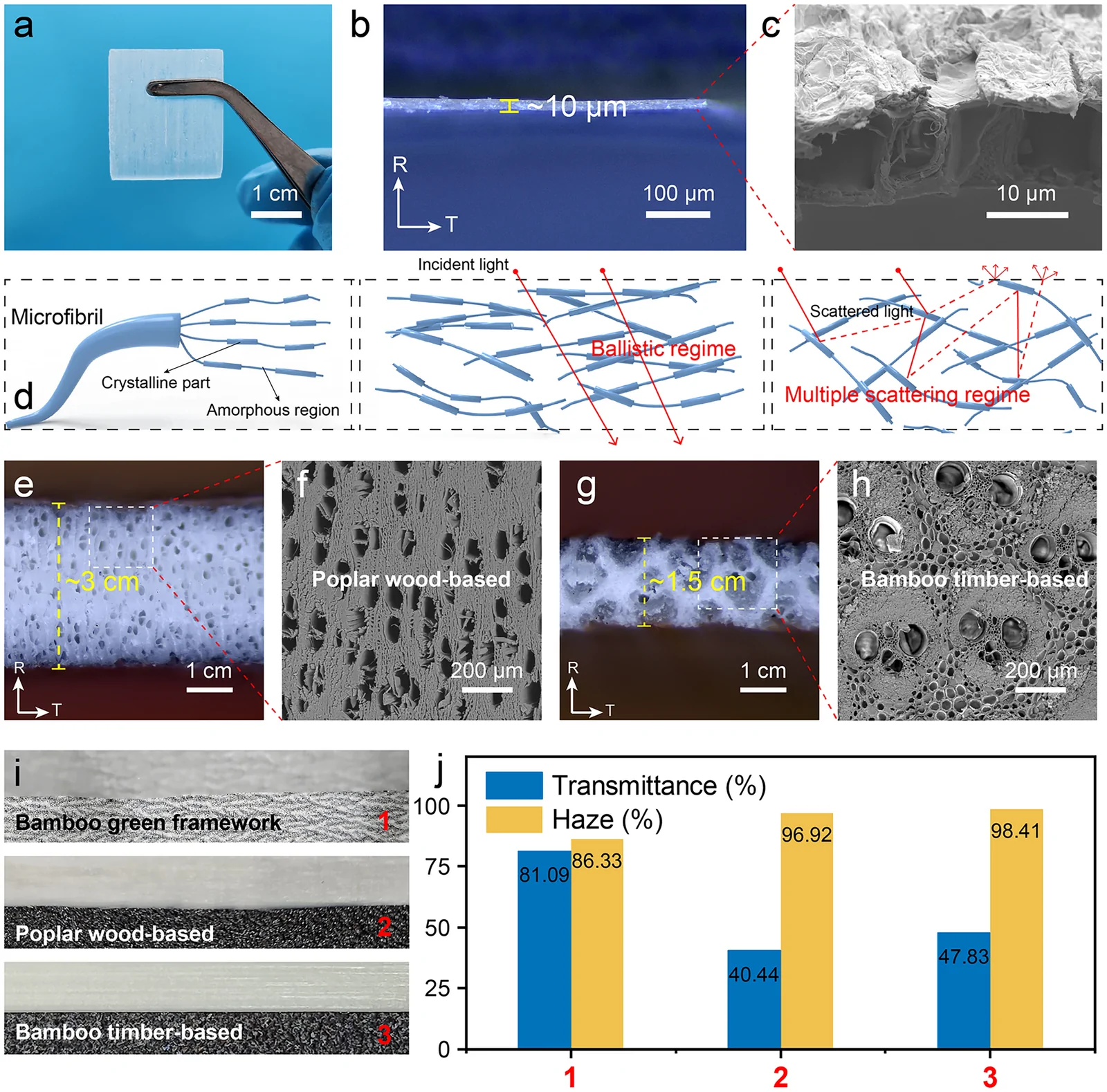
Structural and optical characterization of biomass-derived frameworks. **a–c** Optical and SEM images of BG framework. **d** Schematic of cellulose microfibril architecture and light propagation regimes in the film: ballistic (transparent) versus multiple scattering (hazy). **e–h** Comparative cross sections of **e, f** delignified poplar wood and **g, h** bamboo timber frameworks. **i** Macroscopic transparency demonstration and **j** quantified transmittance/haze at 550 nm for: (1) BG, (2) poplar wood, and (3) bamboo timber frameworks

Native BG-derived frameworks retain the original cellular architecture, displaying distinct stomata on the outer surface and a wrinkled hypodermis-adhered inner layer (Fig. S4). Since the framework consists solely of the epidermal layer and hypodermis, its microscale structure is maintained without mechanical compression, unlike wood- and bamboo timber-derived frameworks. Microstructural characterization (Fig. 4a, b) reveals that the BG framework possesses a highly uniform thickness of 10.0 μm, exhibiting an architecturally optimized monolayer cell wall skeleton (Fig. 4c). When light interacts with the BG framework, multiple optical phenomena occur, including reflection, transmission, refraction, absorption, and scattering [31]. In the ballistic propagation regime (low optical thickness), minimal light interference results in high transparency (81.09% at 550 nm, Fig. 4j). Conversely, multiple scattering induces opacity, as observed in conventional biomass frameworks. In striking contrast, wood- and bamboo timber-based frameworks (Fig. 4e–h) in their uncompressed state display greater thickness (typically 100 times that of BG framework), featuring a stacked multilayer cellular skeleton with inherent opacity (haze > 95%, transmittance < 50% at 550 nm, Fig. 4i, j). Whereas wood/bamboo timber frameworks require thickness reduction to ≤ 100 μm and refractive index matching (e.g., PMMA infiltration) to approach comparable optical performance [36, 37], BG’s monolayer structure intrinsically combines 81.09% transparency and 86.33% haze without these energy-intensive modifications (Fig. 4j).

Energy-dispersive X-ray spectroscopy (EDS) analysis (Fig. S5) revealed distinct elemental distributions on the BG framework surfaces. The outer surface was dominated by C (85.66 wt%) with minimal O (10.90 wt%; O/C ratio: 0.26; Fig. S5a and Table S4), consistent with epidermal waxes (long-chain alkanes) as evidenced by FTIR peaks at 2920 cm^−1^ and 2847 cm^−1^ (CH_2_ stretching vibrations) (Fig. S6a). In contrast, the inner surface exhibited higher O content (43.20 wt%; O/C ratio: 0.76; Fig. S5b and Table S4), matching XPS data (Fig. S6b-d) and predominant C–O (56.12%) and O–C = O (97.6%) bonding states (Table S4), which reflected the presence of cellulose/hemicellulose glycosidic bonds (C–O–C) and carboxyl groups (–COOH). Trace Zn (epidermal enzymes) and Si (hypodermal enrichment, *cf.* Qiu et al. [32]) were detected, with appearance of FTIR peak at 792 cm^−1^ (Si–O stretching vibrations) further confirming Si presence (Fig. S6a). The synergistic effects of Si/wax-induced hydrophobicity and cellulose-driven hydrophilicity endowed the framework with bidirectional wettability (O/C = 0.26 vs. 0.76). This phenomenon is consistent with ultrastructural studies of plant cuticles. The hybrid interface resembles the well-documented “cutin-cellulose sandwich” structure observed in graminaceous plants (e.g., bamboo and maize), where epicuticular waxes (C25-C33 alkanes) coat polysaccharide-rich hypodermis [33]. Similar chemical stratification is evident in *Arabidopsis thaliana* cuticles, with distinct hydrophobic outer and amphiphilic inner layers [34]. Such structural–functional anisotropy highlights the potential for surface-specific functionalization in applications like directional fluid transport or oil–water separation.

**Fig. 5:**
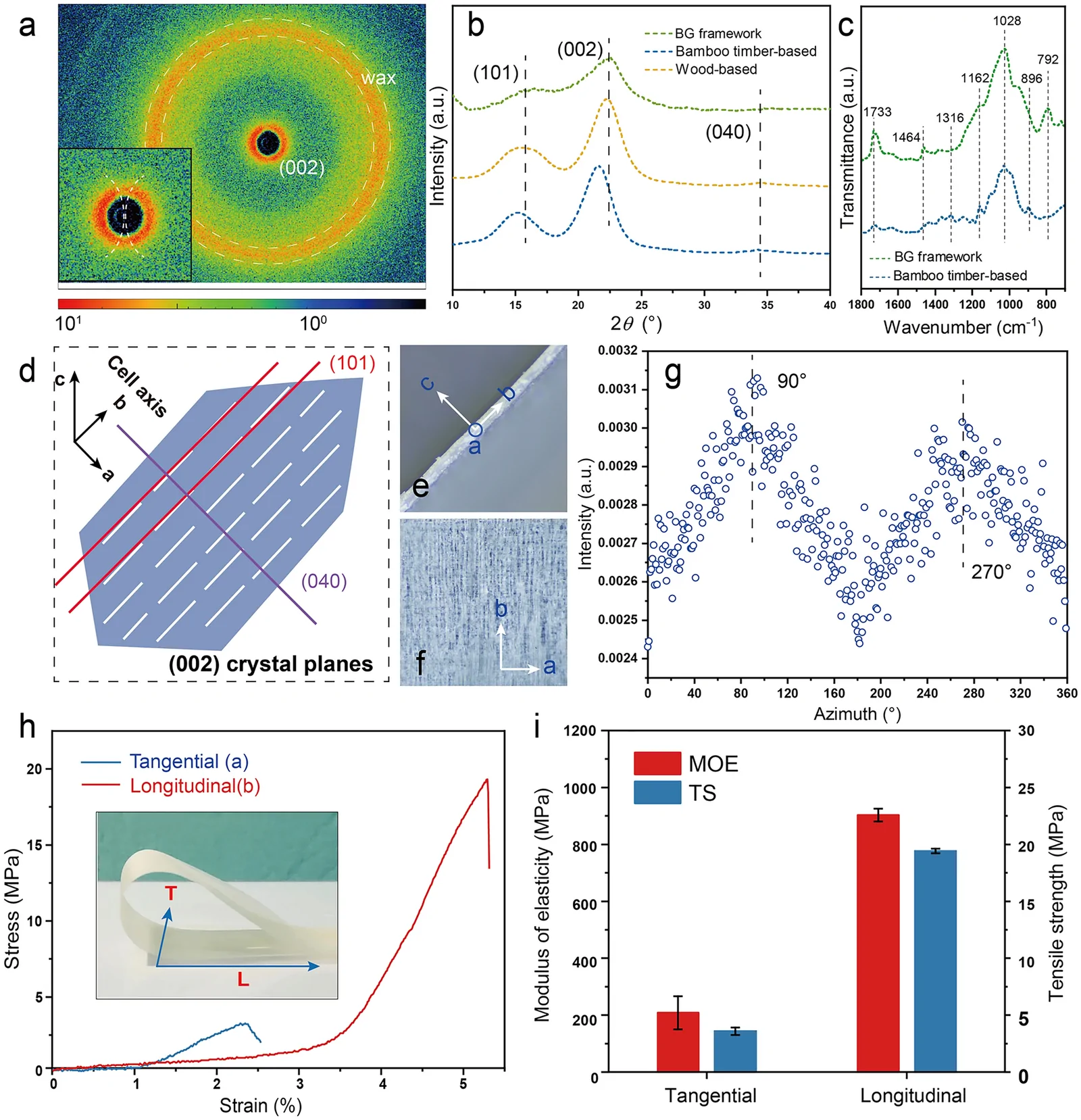
Structural and mechanical characterization of the BG framework. **a** 2D-WAXS scattering pattern. **b** XRD spectra of wood-, bamboo timber-, and BG-based frameworks. **c** FTIR spectra comparing bamboo timber- and BG-based frameworks. **d** Schematic of the relative positions of the (002), (101), and (040) crystallographic planes in cellulose. Orientation diagrams of the BG framework: **e** lateral view, **f** planar view. **g** Azimuthal intensity distribution (I-azimuth) for the (002) crystalline plane. **h** Representative stress–strain curves under tangential and longitudinal loading. **i** Comparative tensile strength and elastic modulus between tangential and longitudinal directions

Quantitative composition analysis (NREL standard, Table S5) identified the framework’s composition as 28.6 wt% cellulose, 3.5 wt% hemicellulose, and 28.3 wt% lignin ratios that preserve the BG cellular structure. Two-dimensional wide-angle X-ray scattering (2D-WAXS) pattern exhibited isotropic crystalline orientation of the framework, as indicated by even-distributed diffraction rings (Fig. 5a). Furthermore, XRD results (Fig. 5b) confirmed the cellulose I crystalline structure, characterized by sharp (101) and (002) diffraction peaks (crystallinity index: 64.76%, Segal–Turley method). While the crystallinity index of the BG framework is lower than that of the bamboo timber framework (84.97%) and wood-based framework (86.13%), this reduced crystallinity allows for greater mobility of cellulose chains in the amorphous regions [35]. However, the FTIR spectrum of BG framework revealed a distinct C = O stretching vibration at 1733 cm^−1^, whereas characteristic saccharide peaks from hemicellulose and cellulose (C–O–C stretching vibrations at 1162 and 896 cm^−1^, O–H in-plane bending vibration at 1316 cm^−1^) [36] are nearly absent compared to the bamboo timber-based framework (Fig. 5c). This results from the shielding effect of wax on cellulose and hemicellulose, as further confirmed by the emergence of an ester C–O–C stretching vibration peak at 1028 cm^−1^ [37]. Consequently, the even-distributed diffraction ring in the 2D-WAXS pattern is likely attributed to wax-mediated amorphous scattering, which obscures the preferential orientation of cellulose crystallites.

To further clarify the orientation characteristics of cellulose, subsequent analysis focused on the (002) crystalline plane: Assuming that the cellulose chains are oriented along the cell axis (*b*-direction), the (002) plane normal (*c*-direction) is perpendicular to the cell axis (*b*), with plane itself parallel to the cell wall plane (*b*-*a* plane) (Fig. 5d). Figure 5e, f confirms the alignment of BG framework cells with the cell axis-*b* (bamboo growth direction), and their wall planes with *b*-*a* planes. The azimuthal scattering intensity distribution (*I*-azimuth) of the (002) plane exhibits two peaks at 90° and 270° (separated by 180°), demonstrating uniaxial vertical symmetry with the *c*-axis perpendicular to *b* (Fig. 5g). Through approximate summation formula $<{\mathrm{cos}}^{2}\varphi >\approx \frac{{\sum }_{i}{\mathrm{cos}}^{2}{\varphi }_{i}\cdot I({\varphi }_{i})}{{\sum }_{i}I({\varphi }_{i})}$ (with azimuthal angle *φ* sampled at 1° intervals and *I*(*φ*) representing the scattering intensity), the calculated Hermans orientation factor *f* = 0.23 suggests that the normal direction of the (002) plane tends to align parallel to the cell wall plane normal (denoted as *c*), demonstrating cellulose chain orientation along *b*. Despite the background elevation in *I*-azimuth from wax scattering (which attenuates peaks), the cellulose’s uniaxial orientation characteristics remain evident in the BG framework. Furthermore, uniaxial tensile tests revealed near-elastic stress–strain behavior of BG framework in the longitudinal direction (b-direction) prior to fracture (Fig. 5h), with an average tensile strength (19.4 MPa) and elastic modulus (903.0 MPa) exceeding tangential values (a-direction) by 5.4 times and 4.3 times, respectively (Fig. 5i). Therefore, clear evidence of a preferred crystalline orientation for cellulose is observed, where the (002) plane is preferentially stacked parallel to the cell wall. This orientational characteristic bridges the nanoscale organization of cellulose microfibrils with the mesoscale architecture and macroscale mechanical properties of the monolayer BG framework.

### Optical Properties and Application of Bamboo Green Framework

**Fig. 6:**
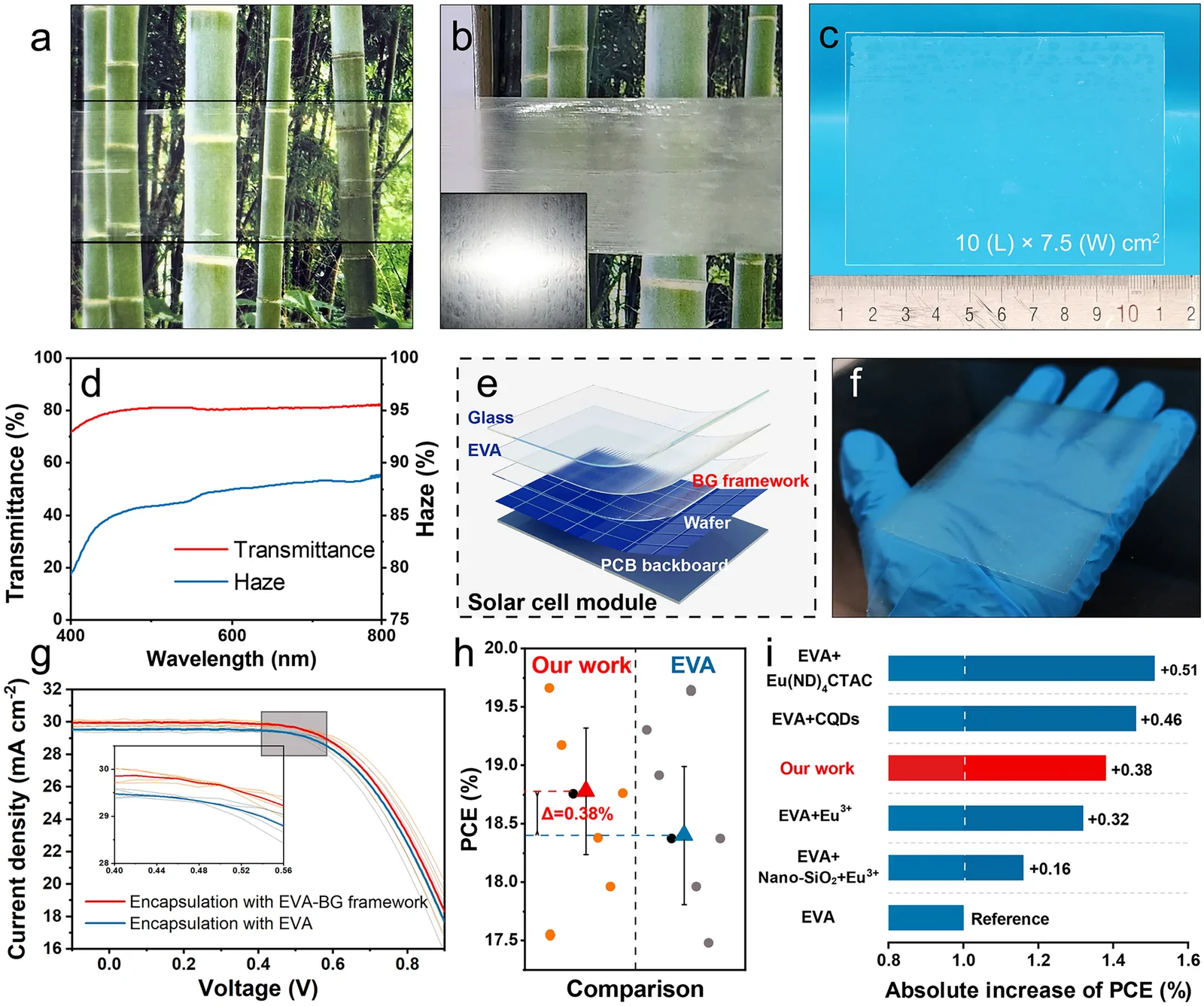
Optical and photovoltaic performance of BG framework. **a** Optical transparency demonstration. **b** Light-scattering demonstration with the framework suspended 5 mm above a color pattern. **c** Macroscale BG framework (10 cm length × 7.5 cm width). **d** Transmittance and haze measurements (400–800 nm). **e** Schematic of BG framework integrated into crystalline silicon solar cells. **f** EVA-encapsulated large-area BG framework. **g** Current–voltage (*J-V*) curves of EVA-only and EVA-BG encapsulated solar cells under AM1.5G illumination. Semi-transparent curves represent five independent replicate measurements for each group, while bold curves show the averaged data. **h** PCE comparison: EVA-BG versus EVA-only encapsulated solar cells. Individual data points (*n* = 5 per group) show replicate measurements, with error bars indicating standard deviation. The dashed line marks the mean PCE enhancement (0.41% absolute). **i** PCE enhancement benchmarking in crystalline silicon photovoltaics: EVA-BG encapsulated devices versus modified EVA-encapsulated references. [44–49]

Optical characterization via integrating sphere UV/visible spectrophotometer showed > 80% transmittance at *λ* > 450 nm (Fig. 6a, d), attributed to the framework’s microscale thickness and short optical path (Fig. 4c). Haze reached 80–88% across the visible spectrum (400–800 nm), demonstrating strong light scattering (Fig. 6d). When placed 5 mm above a Moso bamboo image, the framework blurred the pattern, further confirming its high haze (Fig. 6b). The wax-cellulose hybrid interface (O/C = 0.26 vs. 0.76, Table S4) creates Mie scattering centers without sacrificing transparency, unlike nanoparticle-doped polymers. As summarized in Table 3, the BG framework achieves 88% haze and 80% transparency at merely 10 μm thickness—a performance unattainable by wood or bamboo timber at 100 × greater thickness. While nanocellulose films offer higher transparency (90%), their energy-intensive production (e.g., TEMPO oxidation) and isotropic restacking sacrifice the natural hierarchical porosity essential for broadband light scattering. Notably, the wax-preserved cellulose alignment in BG (Hermans factor: 0.23) provides a high tensile modulus of 903 MPa, avoiding the brittleness of thick wood frameworks while eliminating the need for synthetic binders required in nanocellulose composites. These properties make it promising for optoelectronics, particularly in light-coupling applications (e.g., solar cells), where it may boost photovoltaic efficiency.

**Table 3:** Comparative performance of cellulose-based frameworks

Material	Thickness (μm)	Haze (%)	Transparency (%)	Tensile modulus (MPa)	Key limitations	References
Bamboo green (This work)	10	< 88	< 80	903	Limited to monolayer structure	
Delignified wood	1000–2000	< 85	< 40	400–700	Requires compression for transparency	[38, 39]
Delignified bamboo timber	600–1500	< 80	< 50	600–900	High opacity at usable thickness	[40, 41]
Nanocellulose film	20–50	< 70	< 90	2000–3000	Energy-intensive production	[42, 43]
Synthetic composite	50–100	< 60	< 90	1000–1500	Non-biodegradable polymer binder	[15, 16]

All optical properties were measured under standard conditions across 400–800 nm wavelength range

An internode bamboo culm (10 cm height × 8.4 cm diameter) was processed in this work, yielding a total framework area of 270 cm^2^ (Figs. 6c and S7)—surpassing standard silicon wafer dimensions (maximum 243.4 cm^2^; 15.6 cm × 15.6 cm). This value reflects the physical dimensions of the raw material rather than statistical averaging, as the peeling process maintains structural continuity of the BG framework. The high-haze transparent cellulose framework functions as an eco-friendly anti-reflective coating, enhancing solar cell performance through forward light scattering (haze: 80%–88%), minimizing reflection losses and improving PCE. When encapsulated with ethylene–vinyl acetate (EVA)—a low-photodegradability polymer widely used in photovoltaics (Fig. 6e, f)—the framework boosted PCE by 0.41% absolute (18.74% → 19.15%, Fig. 6g, h and Table S6) in polycrystalline silicon cells (12 cm^2^). The reported PCE improvement was validated through 3 independent devices, 5 measurements per device (15 datasets total), and obtained statistical significance *p* = 0.018 < 0.05 with large effect size *η*^2^ = 0.489 > 0.20 (one-way ANOVA, Table S6). This outperforms conventional Eu^3+^-doped EVA layers (Fig. 6i) [44–49]. The framework’s sustainability, low-cost processing, and environmental compatibility further support its viability for photovoltaic applications.

### Bamboo Green’s Hierarchical Structure Enables Sustainable Biomass Valorization

Bamboo green (BG) exhibits a unique hierarchical structure distinct from bamboo timber or wood. While the latter are dominated by densely packed fiber bundles or fiber cells (Fig. S8), BG comprises 3–4 layers of parenchyma-rich cortex beneath a wax-impregnated epidermis (Fig. 2a, b). This architecture offers two critical advantages: (i) The thin-walled parenchyma cells (20–30 μm diameter, Fig. S1) provide natural pathways for chemical penetration (71% surface porosity vs. < 50% in fiber bundles), enabling efficient HCOOOH-mediated peeling; and (ii) the epidermal wax layer (85.66 wt% C, Fig. S5a) shields underlying cellulose fibrils from acid degradation, preserving native crystallinity (64.76%, XRD in Fig. 5b). In contrast, the inherent fiber-composite architecture of wood and bamboo timber necessitates frameworks with millimeter-scale thicknesses, resulting in low transparency (< 40%) due to increased light scattering.

The wax-cellulose hybrid interface in BG frameworks plays a pivotal role in optimizing optical and mechanical properties. FTIR and XPS analyses (Fig. S6) confirm that epidermal waxes (C–H stretching at 2920 and 2847 cm⁻^1^) form a hydrophobic outer layer (O/C = 0.26), while the inner surface retains hydrophilic cellulose (O/C = 0.76). This bidirectional wettability mitigates interfacial reflection losses, contributing to the 88% haze with 80% transparency. Moreover, WAXS reveals that wax coatings partially obscure cellulose’s preferential orientation (Hermans factor: 0.23), yet the underlying fibril alignment along the bamboo growth axis (*b*-direction) ensures high longitudinal tensile strength (903 MPa, 4.3 × higher than tangential). Such anisotropic properties are also exists in isotropic natural wood- or bamboo timber-based frameworks, which resulting from fiber cells or fiber bundles orientation.

From a circular economy perspective, BG represents an underutilized industrial by-product. Current bamboo processing discards BG due to its waxy surface, which impedes adhesive bonding in composites. This work transforms the waste into high-value optical materials—270 cm^2^ of framework per bamboo culm (Fig. S7), sufficient to cover standard silicon wafers (maximum 243.4 cm^2^). The HCOOOH peeling process (MLR: 7.4%) consumes minimal reagents compared to nanocellulose production, while the preserved wax layer eliminates the need for synthetic hydrophobic coatings. This “waste-to-optical-material” paradigm aligns with global demands for sustainable manufacturing, offering a scalable route to decarbonize optoelectronic supply chains.

## Conclusion

Inspired by the hierarchical structure of squid skin, this work developed a biomimetic peeling strategy using peroxyformic acid (HCOOOH) to isolate intact 10-μm-thick bamboo green (BG) cellulose frameworks. This process selectively targets parenchyma cell junctions through cleavage of hydrogen and phenyl glycoside bonds, while preserving the native cellulose I crystallinity (64.76%) and uniaxial fibril alignment (Hermans factor: 0.23). The BG framework achieves an unprecedented combination of 88% haze and 80% transparency, surpassing delignified wood/bamboo timber and rivaling synthetic composites. Its anisotropic mechanical properties—longitudinal tensile strength and modulus 5.4 × and 4.3 × higher than tangential values, respectively—enable robust integration into optoelectronic devices. As a light-management layer in polycrystalline silicon solar cells, the BG framework delivers a 0.41% absolute PCE enhancement (18.74% → 19.15%), outperforming conventional anti-reflective coatings. The monolayer cellulose-wax-Si hybrid architecture further enables multifunctional applications, from hydrophobic surfaces to silicon-carbon composites. This work transforms bamboo green—an industrial by-product—into high-performance optical materials through a scalable process (270 cm^2^ framework per culm). The waste-to-wealth approach aligns with circular bioeconomy principles, offering a sustainable alternative to energy-intensive nanocellulose production.

## Supplementary Information

Below is the link to the electronic supplementary material.

Supplementary file1 (DOCX 4063 KB)
