# Supplementary material for: Bioinspired Precision Peeling of Ultrathin Bamboo Green Cellulose Frameworks for Light Management in Optoelectronics
Source: Nanomicro Lett. 2025 Aug 5;18:19. doi: 10.1007/s40820-025-01867-1 (PMC12325841; doi:10.1007/s40820-025-01867-1)
Supplement: Supplementary file 1 — Supplementary file1 (DOCX 4063 KB) [file 40820_2025_1867_MOESM1_ESM.docx]

Supporting Information for

**Bioinspired Precision Peeling of Ultrathin Bamboo Green Cellulose Frameworks for Light Management in Optoelectronics**

Yan Wang^1,#^, Yuan Zhang^1,#^, Yingfeng Zuo^1,^*, Dawei Zhao^2^, Yiqiang Wu^1,^*

^1^ College of Materials and Energy, Central South University of Forestry and Technology, Changsha, Hunan 410004, P. R. China

^2^ Key Laboratory on Resources Chemicals and Materials of Ministry of Education, Shenyang University of Chemical Technology, Shenyang, Liaoning 110142, P. R. China

^#^Yan Wang and Yuan Zhang contributed equally to this work.

*Corresponding authors. E-mail: [zuoyf1986@163.com](mailto:zuoyf1986@163.com) (Yingfeng Zuo); [wuyq0506@126.com](mailto:wuyq0506@126.com) (Yiqiang Wu)

**Supplementary Figures and Tables**

**
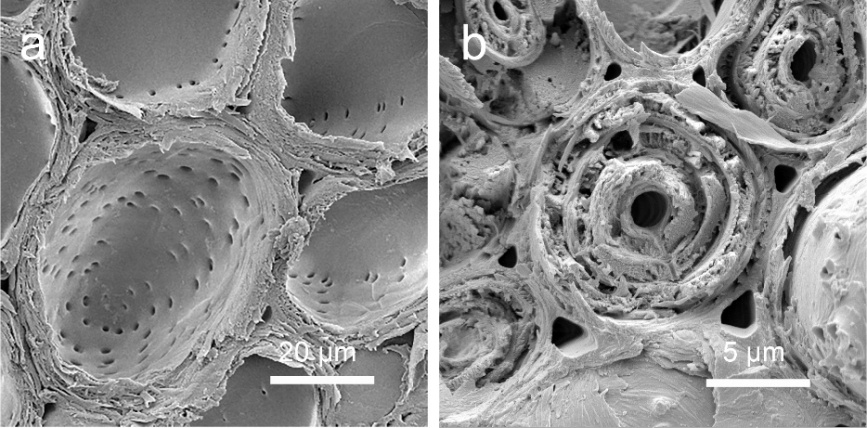
**

**Fig. S1** SEM cross-sections: (**a**) parenchyma cells (~71% surface porosity), (**b**) vascular bundles (50.5–20.3% surface porosity). Surface porosity data are from reference [S1]

The parenchyma cells (a) display significantly higher porosity (~71%) compared to vascular bundles (b, 50.5–20.3%), attributed to their characteristic thin walls and abundant pit structures (red arrows) that create interconnected penetration pathways. This distinct morphological difference enables selective chemical access during the peeling process, where reagents preferentially infiltrate through the porous parenchyma network while sparing the denser vascular regions.

**

**

**Fig. S2** Temperature-time profiles of reaction solutions with varying H_2_O_2_:HCOOH molar ratios

The temperature-time profiles reveal distinct reaction behaviors for different H_2_O_2_:HCOOH molar ratios at 60 °C. While solutions with 1.5:1, 1:1, and 1:2 ratios exhibited secondary temperature increases (98 °C max) due to exothermic peroxyformic acid (HCOOOH) formation, the 2:1 ratio solution showed no exotherm beyond 60 °C, indicating negligible HCOOOH generation. Consistent with this observation, bamboo samples treated with the 2:1 ratio solution displayed no peeling behavior, confirming the essential role of HCOOOH in the peeling process. The 1:1 H_2_O_2_:HCOOH ratio maximizes active HCOOOH generation while minimizing parasitic decomposition (Fig. S2 thermal data). Kinetic studies show this ratio achieves optimal balance between: Reaction exothermicity (ΔT=38 °C, peak at 98 °C); Deviation from 1:1 either reduces yield (low conversion at 2:1) or increases byproducts (formate esters at 1:2).


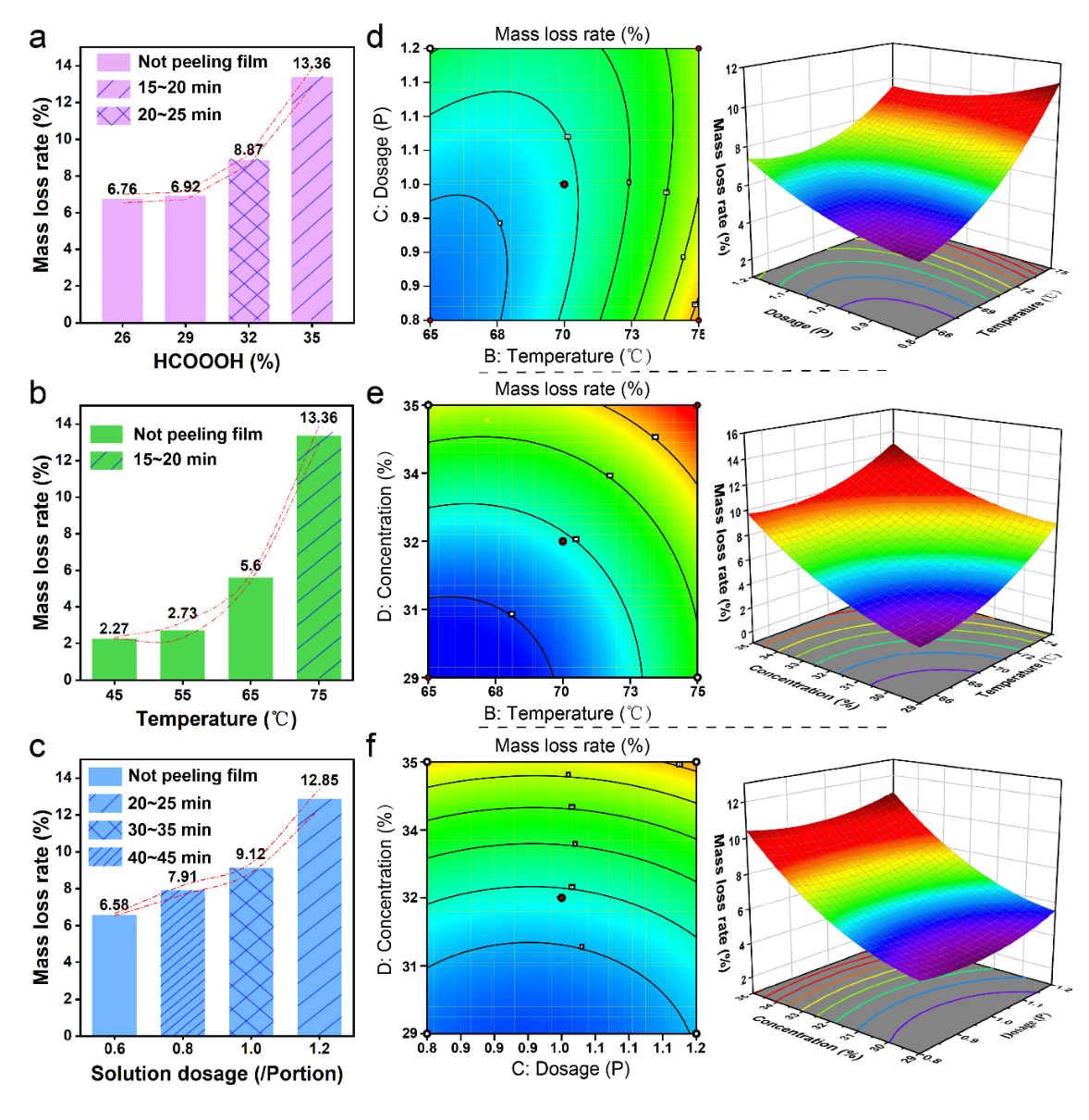


**Fig. S3** Process optimization results from single factor design and RSM. Effect of (**a**) HCOOOH concentration, (**b**) reaction temperature, and (**c**) HCOOOH dosage on peeling time and mass loss rate. (**d-f**) Interaction effects shown by contour plots and response surfaces: (**d**) temperature-B vs. dosage-C, (**e**) temperature-B vs. concentration-D, and (**f**) dosage-C vs. concentration-D

The response surface analysis reveals key process interactions for bamboo green (BG) peeling. Steeper response surfaces and elliptical contours (d-f) indicate stronger factor interactions. Reagent dosage (C) and temperature (B) show the most significant combined effect on mass loss rate (*MLR*). The target *MLR* range (7%–13.35%) ensures complete BG framework detachment from the bamboo strip. Lower *MLR* (<7%) results in incomplete peeling, while higher *MLR* (>13.35%) causes over-degradation of the bamboo strip. These parameters guarantee efficient BG framework isolation while minimizing biomass waste (Contour plots provide visual guidance for parameter selection; validated optimal conditions in Table 2).

**
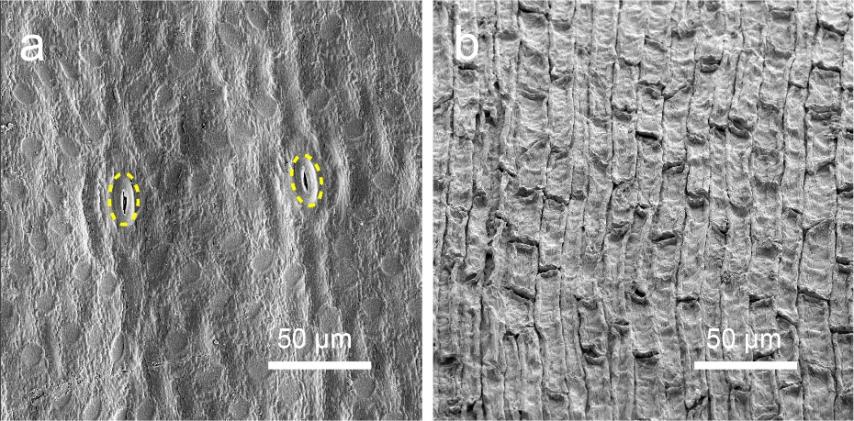
**

**Fig. S4** SEM images of the BG framework: (**a**) outer and (**b**) inner surfaces. Stomata (yellow circles) distributed across the outer surface of BG framework

**
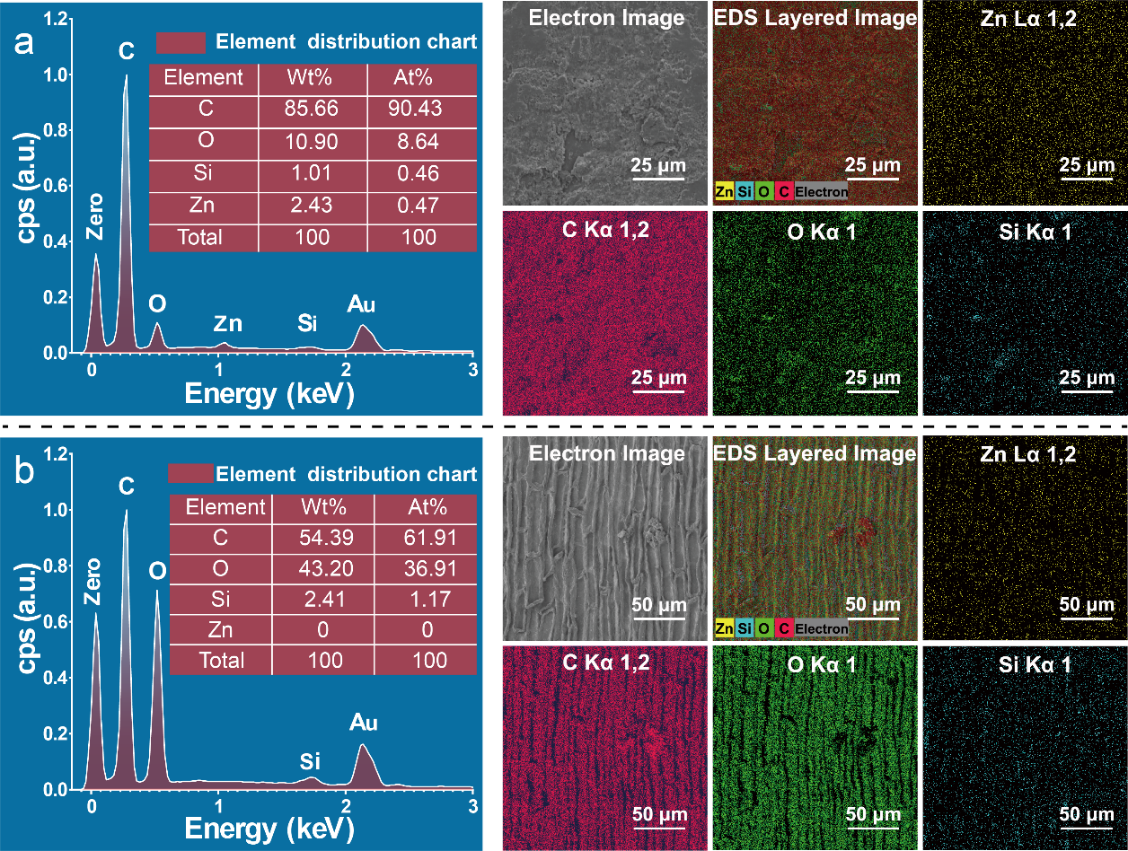
**

**Fig. S5** Elemental composition analysis by EDS: (**a**) outer surface and (**b**) inner surface of BG framework


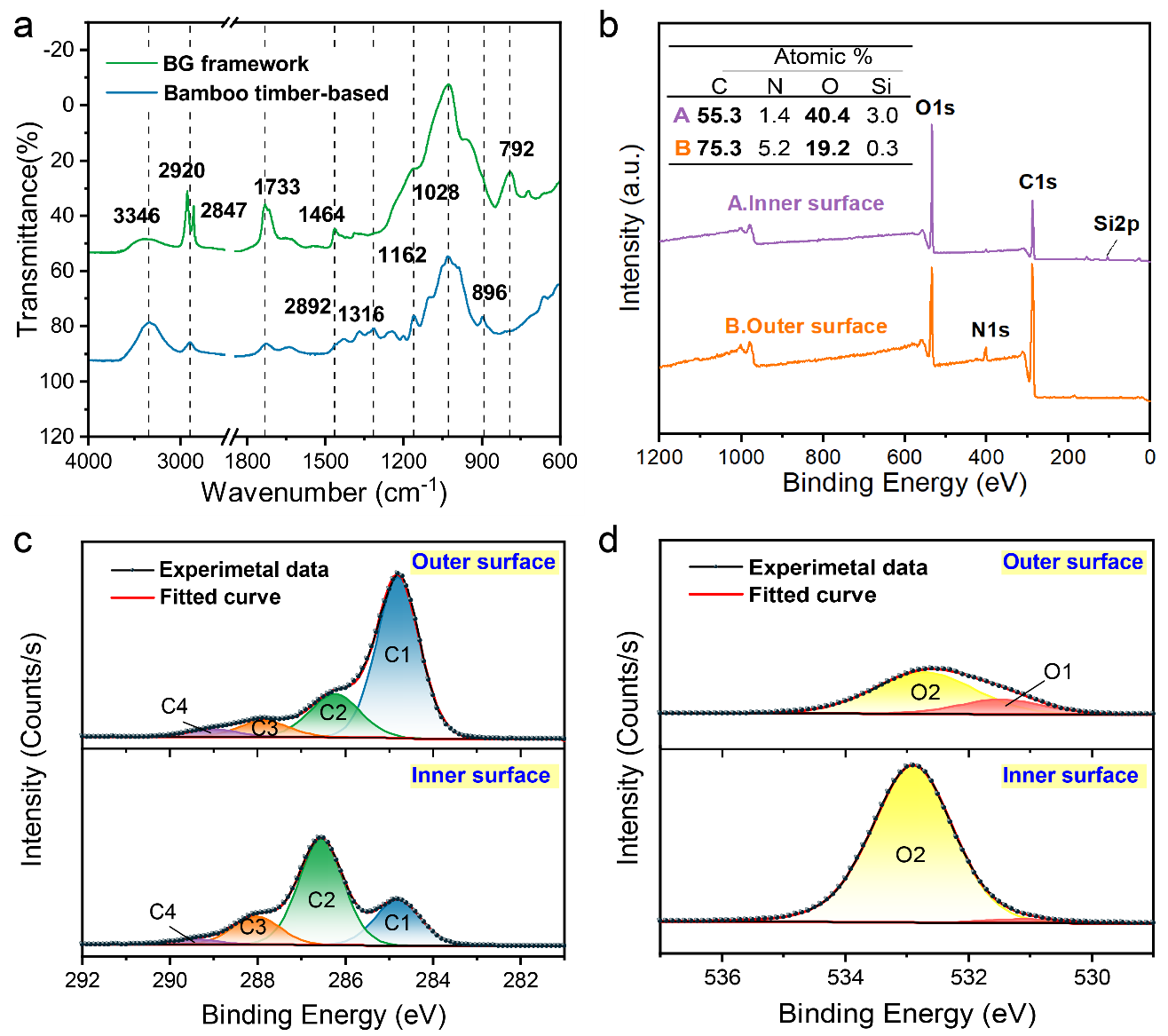


**Fig. S6** Surface chemistry analysis of BG framework. (**a**) FTIR spectra comparing the BG framework and bamboo timber-based framework. (**b**) XPS survey spectra of the outer and inner surfaces of the BG framework. High-resolution XPS spectra of (**c**) C 1s and (**d**) O 1s from the outer and inner surfaces


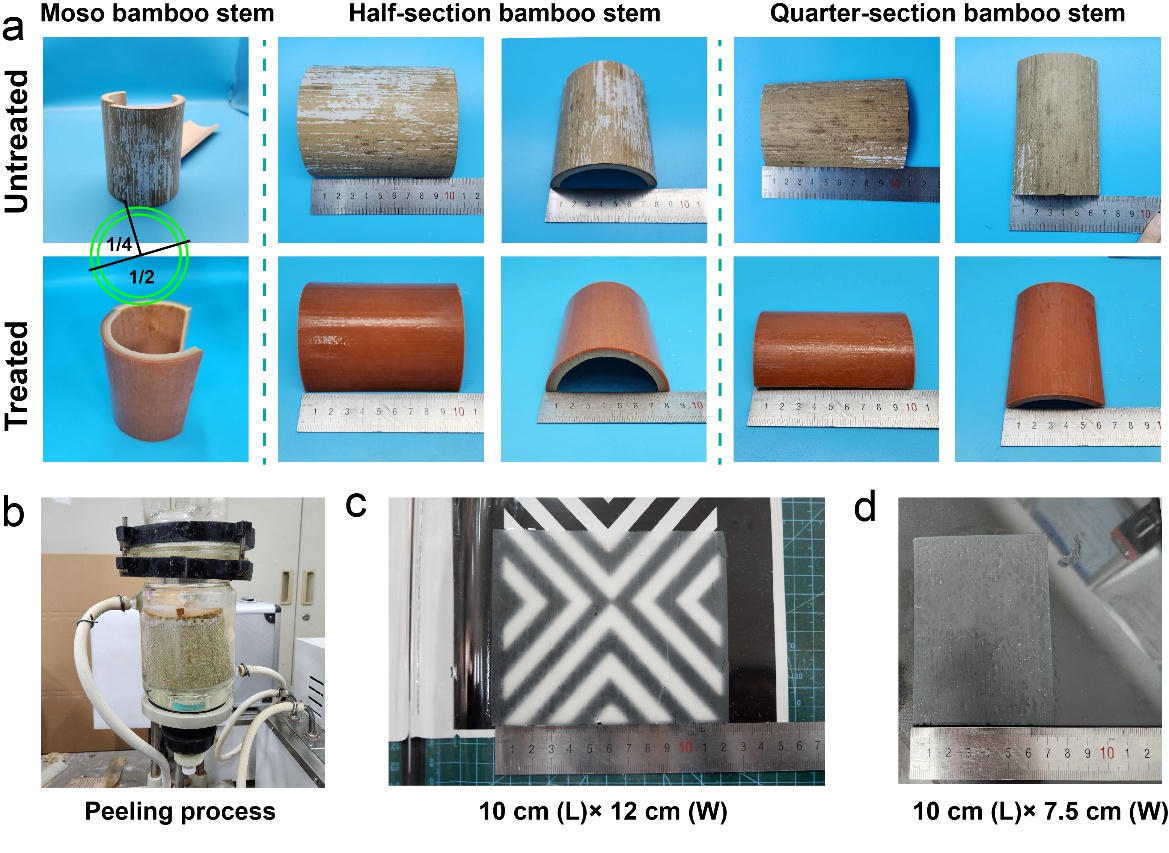


**Fig. S7** Bamboo stem peeling process and resulting BG framework. (**a**) Half and quarter bamboo stems before and after peeling. (**b**) Equipment used for the peeling process. (**c, d**) Photographs of the peeled BG framework, with dimensions of (**c**) 10 cm (length) × 12 cm (width) and (**d**) 10 cm (length) × 7.5 cm (width)

A single internode bamboo stem with dimensions of 10 cm (height) × 8.4 cm (diameter) and 0.8 cm wall thickness was radially dissected into 1/2 and 1/4 sections for peeling experiments. The experiment utilized a 1 L high-temperature water-circulating glass reactor as the reaction vessel. Morphological changes of bamboo sections before and after BG peeling are shown in panel (a). The resulting BG frameworks exhibit two size variants: 10 cm (length) × 12 cm (width) (c); 10 cm (length) × 7.5 cm (width) (d). A single bamboo stem yields up to 270 cm^2^ (120 cm^2^ + 75 × 2 cm^2^) of BG framework, exceeding the maximum size (225 cm^2^) of commercial solar cells (Kyocera). The framework dimensions can be further customized through strategic node positioning during stem selection and controlled peeling process parameters. This enables modular assembly of frameworks to match various photovoltaic requirements.


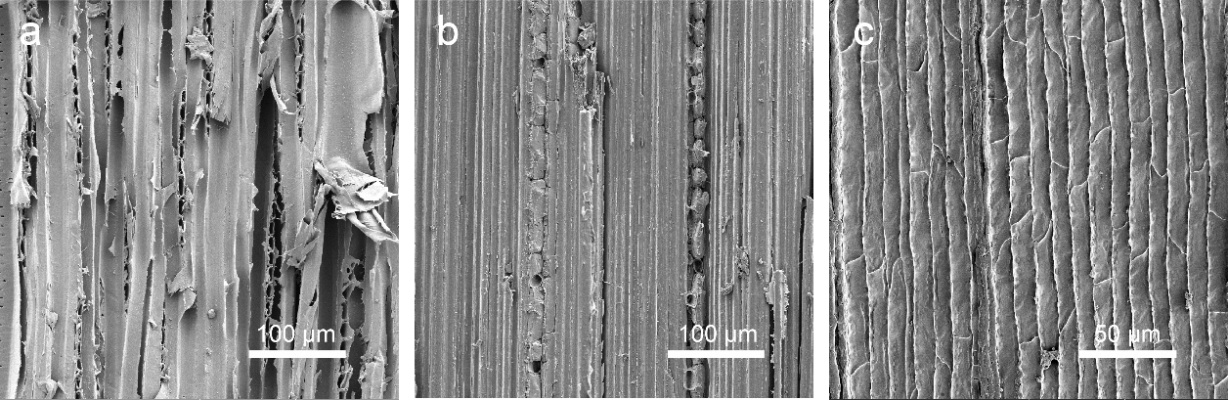


**Fig. S8** Structural comparison showing the directional organization of (**a**) wood (parallel fiber cells), (**b**) bamboo timber (aligned fiber bundles), and (**c**) bamboo green (longitudinally arranged epidermal cells)

**Table S1** XPS spectral parameters of untreated and treated bamboo samples

| Bamboo samples | O/C  atomic ratios | C（%） | | | | O（%） | | | |
| --- | --- | --- | --- | --- | --- | --- | --- | --- | --- |
|  |  | C1 | C2 | C3 | C4 | | O1 | O2 | O3 |
| Untreated | 0.38 | 42.59 | 45.77 | 6.67 | 4.97 | | 6.55 | 84.68 | 8.77 |
| Treated | 0.55 | 35.16 | 46.92 | 12.89 | 5.02 | | 4.85 | 91.79 | 3.36 |

(C1: C-C/C-H; C2: C-O; C3: O-C-O /C=O; C4: O-C=O; O1: C-OH/C=O; O2: O-C=O; O3: O=C-O.)

**Table S2** Chemical composition of bamboo samples determined by NREL standard method

| Composition | Bamboo samples | |
| --- | --- | --- |
|  | Untreated | Treated |
| Cellulose | 31.63 ± 0.48 wt% | 31.03 ± 1.39 wt% |
| Hemicellulose | 8.00 ± 0.67 wt% | 4.0 ± 0.59 wt% |
| Lignin | 38.67 ± 0.54 wt% | 37.73 ± 0.86 wt% |

**Table S3** Factors and levels in the Box-Behnken Design (BBD)

| Factor  Level | B | C | D |
| --- | --- | --- | --- |
|  | Temperature (°C) | Dosage (P, Portion) | Concentration (%) |
| Level (–1) | 65 | 0.8 | 29 |
| Level (0) | 70 | 1.0 | 32 |
| Level (+1) | 75 | 1.2 | 35 |

**Table S4** XPS spectral parameters of the outer and inner surfaces of BG framework

| BG framework | O/C atomic Ratios | C（%） | | | O（%） | | |
| --- | --- | --- | --- | --- | --- | --- | --- |
|  |  | C1 | C2 | C3 | C4 | O1 | O2 |
| Inner surface | 0.76 | 24.88 | 56.12 | 16.05 | 2.95 | 2.40 | 97.60 |
| Outer surface | 0.26 | 67.27 | 21.15 | 7.94 | 3.64 | 23.26 | 76.74 |

(C1: C-C/C-H; C2: C-O; C3: O-C-O /C=O; C4: O-C=O; O1: C-OH/C=O; O2: O-C=O; O3: O=C-O.)

**Table S5** Chemical composition of BG framework determined by NREL standard method

| Composition | Content | 1 | 2 | 3 |
| --- | --- | --- | --- | --- |
| Cellulose | 28.6 ± 0.21 wt% | 28.9 wt% | 28.4 wt% | 28.6 wt% |
| Hemicellulose | 3.5 ± 0.19 wt% | 3.6 wt% | 3.2 wt% | 3.6 wt% |
| Lignin | 28.3 ± 0.25 wt% | 28.2 wt% | 28.0 wt% | 28.6 wt% |

**Table S6** One-way ANOVA results of photoelectric conversion efficiency (PCE)

| PCE (%) | Cell Group | 1 | | 2 | | | 3 | | | 4 | | 5 |
| --- | --- | --- | --- | --- | --- | --- | --- | --- | --- | --- | --- | --- |
|  | EVA-BG | 19.5048 | | 19.1718 | | | 19.0487 | | | 18.9225 | | 19.0911 |
|  | EVA-only | 18.7448 | | 18.9132 | | | 18.4680 | | | 18.9864 | | 18.5784 |
|  | Unprocessed | 19.0827 | | 18.4769 | | | 18.7087 | | | 18.5715 | | 18.6111 |
| Cell Group | Mean (%) | Std. Deviation | | | Mean square | | | *F* | *p*-value | | Effect Size | |
| EVA-BG | 19.1478 | 0.2346 | | | 0.284 | | | 5.734 | 0.018 < 0.05, **significance** | | η² = 0.489 > 0.20 | |
| EVA-only | 18.7382 | 0.2056 | | |  |  |  |  |  |  |  |  |
| Unprocessed | 18.6900 | 0.2362 | | |  |  |  |  |  |  |  |  |
| Result verification | | | | | | | | | | | | |
| Levene | *F* = 0.128 | | *p*-value = 0.881 | | | Equal variances assumed | | | | | | |
| Tukey HSD | MD = 0.4096 | | Std. Error = 0.1410 | | | p-value = 0.027 < 0.05, **significance**  (EVA-BG vs. EVA-only) | | | | | | |

**Supplementary References**

1. X. Yang, X. Pang, X. Liu, S. Yang, X. Li, Determining the pore structure and radial variability of moso bamboo (Phyllostachys edulis). Wood Sci. Technol. **57**, 345–357 (2022). <https://doi.org/10.1007/s00226-022-01451-5>
